# Supplementary material for: Divergent changes in particulate and mineral-associated organic carbon upon permafrost thaw
Source: Nat Commun. 2022 Aug 29;13:5073. doi: 10.1038/s41467-022-32681-7 (PMC9424277; doi:10.1038/s41467-022-32681-7)
Supplement: Supplementary file 1 — Supplementary Information [file 41467_2022_32681_MOESM1_ESM.pdf]

Supplementary Information for

Divergent changes in particulate and mineral-associated organic carbon upon permafrost thaw

Liu et al.

Supplementary Figures

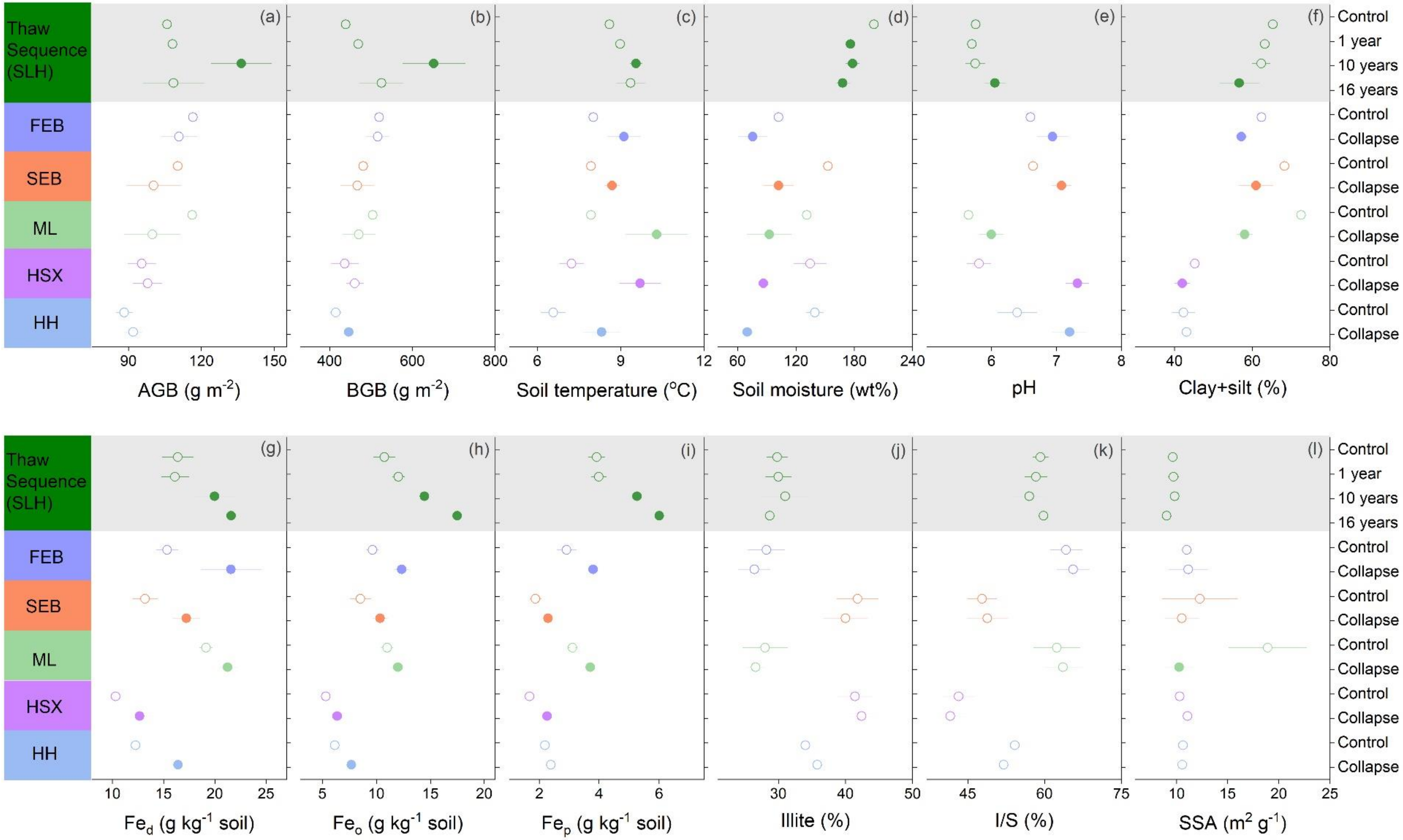

Supplementary Fig. 1. Comparisons of biotic and abiotic factors between collapsed and control plots across the Tibetan thermokarst-impacted sites. a-l, Changes in AGB (a), BGB (b), soil temperature (c), soil moisture (d), pH (e), soil texture (f), Fe_d (g), Fe_o (h), Fe_p (i), illite (j), I/S (k) and SSA (l) induced by permafrost collapse. SLH, site at Shaliuhe; FEB, the first site at Ebo; SEB, the second site at Ebo; ML, site at Mole; HSX, site at Huashixia; HH, site at Huanghe; AGB, aboveground biomass; BGB, belowground biomass; Clay + silt, the percentage of clay and silt; Fe_d, pedogenic Fe oxides; Fe_o, poorly crystalline Fe oxides; Fe_p, organically complexed Fe oxides; I/S, mixed-layer illite/smectite minerals; SSA, specific surface area in mineral-associated organic matter. The shadings indicate the parameters within the permafrost thaw sequence at SLH site. The dots indicate the mean value of each independent variable, and closed circles signify statistical difference from control plot (non-collapse) within each parameters (LSD test, $P < 0.05$), and the error bars represent 95% confidence intervals. These parameters involving the vegetation and soil physicochemical properties along the thaw sequence and across the regional sites were measured in 2014 and 2020, respectively. Part of biotic and abiotic parameters along the thaw sequence, such as aboveground and belowground biomass, soil temperature, moisture, pH and soil texture, was reanalyzed from published data in refs.[1,2], while parameters across the five additional sites at the regional scale were measured in this study.

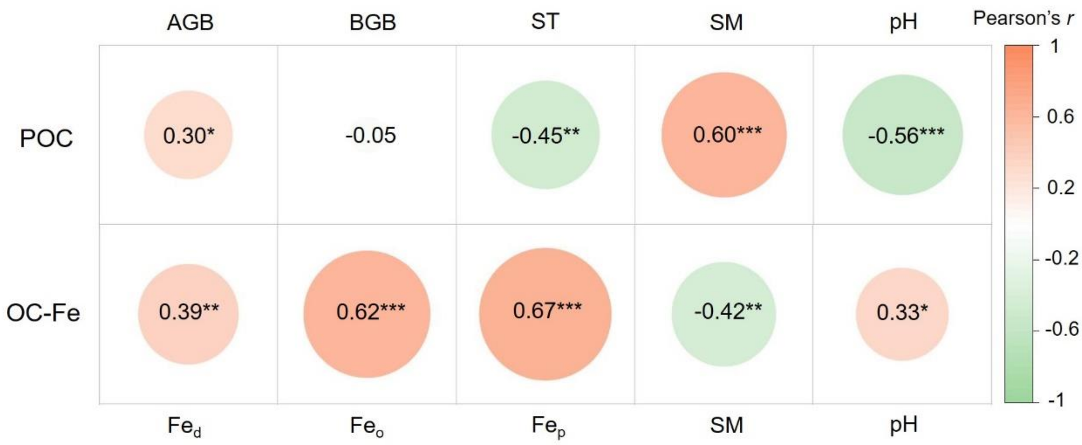

Supplementary Fig. 2. Relationships of the contents of POC and OC-Fe with potential factors along the thaw sequence. POC, particulate organic carbon; OC-Fe, iron-bound organic carbon; AGB, aboveground biomass; BGB, belowground biomass; ST, soil temperature; SM, soil moisture; Fe_d, pedogenic Fe oxides; Fe_o, poorly crystalline Fe oxides; Fe_p, organically complexed Fe oxides. The color indicates the direction and strength of the association between response and explained variables. *$P < 0.05$, **$P < 0.01$, and ***$P < 0.001$.

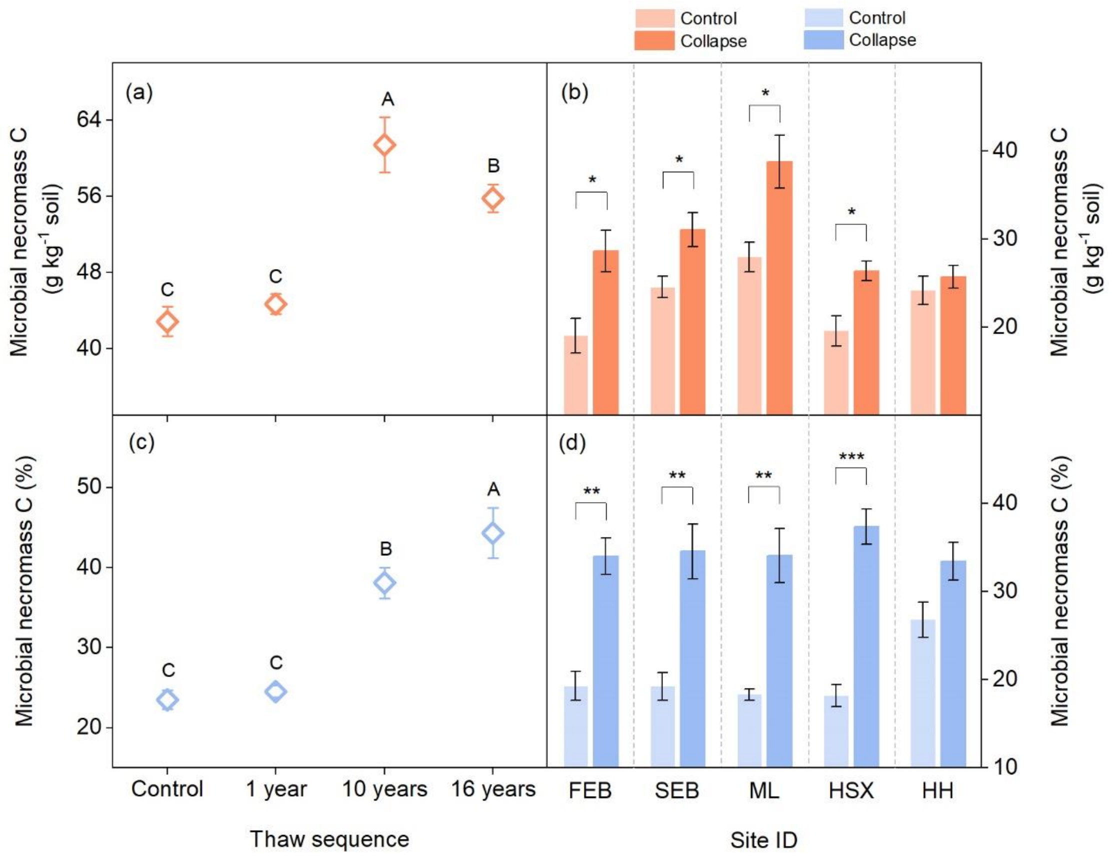

Supplementary Fig. 3. Changes in microbial necromass carbon content and its proportion to bulk soil carbon induced by thermokarst formation. a-b, Changes in microbial necromass carbon content along the thaw sequence (a) and at the five additional sites over the regional scale (b). c-d, Shifts in the proportion of microbial necromass carbon to bulk soil carbon along the thaw sequence (c) and at the regional thermokarst-impacted sites (d). FEB, the first site at Ebo; SEB, the second site at Ebo; ML, site at Mole; HSX, site at Huashixia; HH, site at Huanghe. Error bars represent standard errors. Different capital letters indicate significant differences for the variables within plots along the thaw sequence (LSD test, $P < 0.05$). Dashed lines distinguish different thermokarst-impacted sites, denoting that the parameters are compared between collapsed and control plots in each site rather than across various study sites. *$P < 0.05$, **$P < 0.01$, and ***$P < 0.001$.

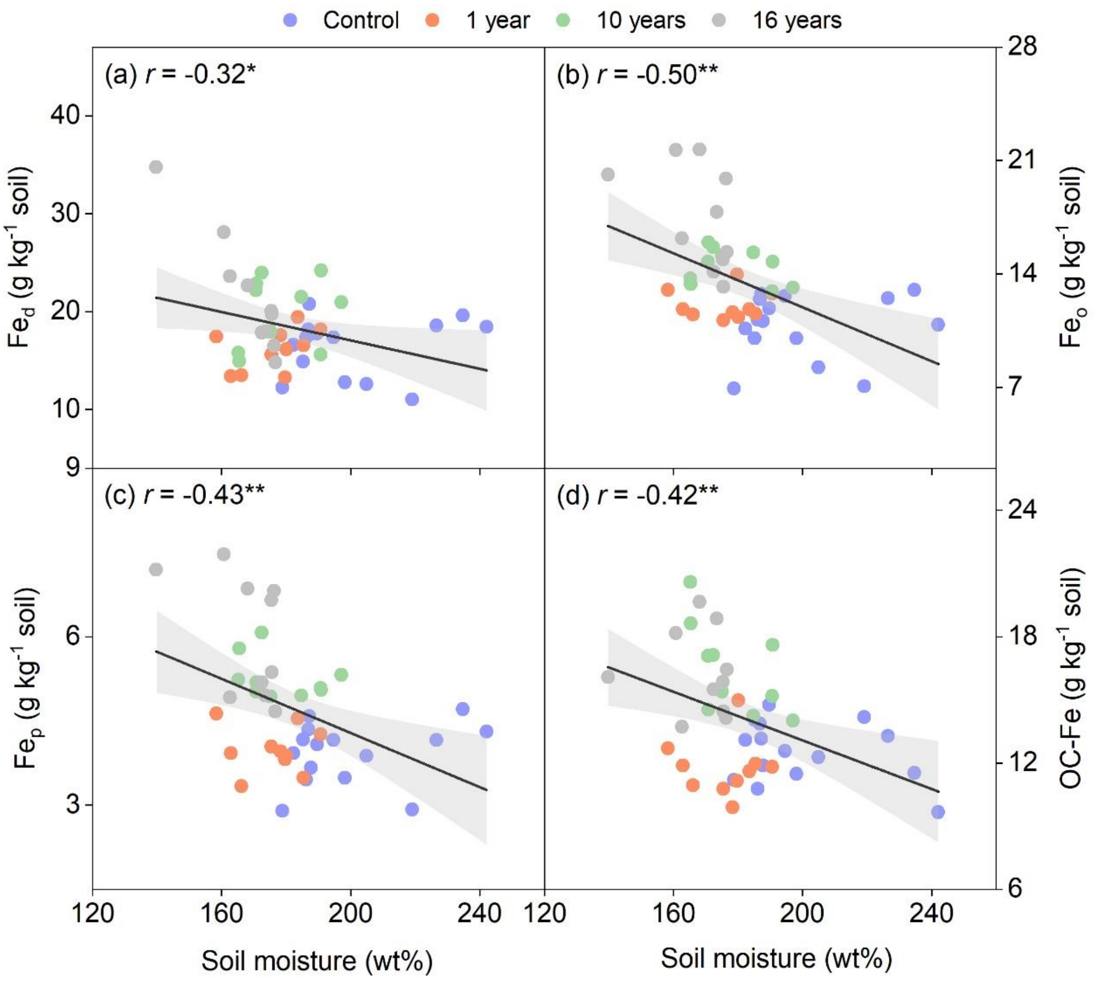

Supplementary Fig. 4. Relationships of the contents of Fe oxides and OC-Fe with soil moisture along the thaw sequence. a-d, Linkages of Fe_d (a), Fe_o (b), Fe_p (c) and OC-Fe (d) with soil moisture. Fe_d, pedogenic Fe oxides; Fe_o, poorly crystalline Fe oxides; Fe_p, organically complexed Fe oxides; OC-Fe, iron-bound organic carbon. The linear regression lines with 95% confidence intervals represent the predicted effects of fixed factors. **P* < 0.05 and ***P* < 0.01.

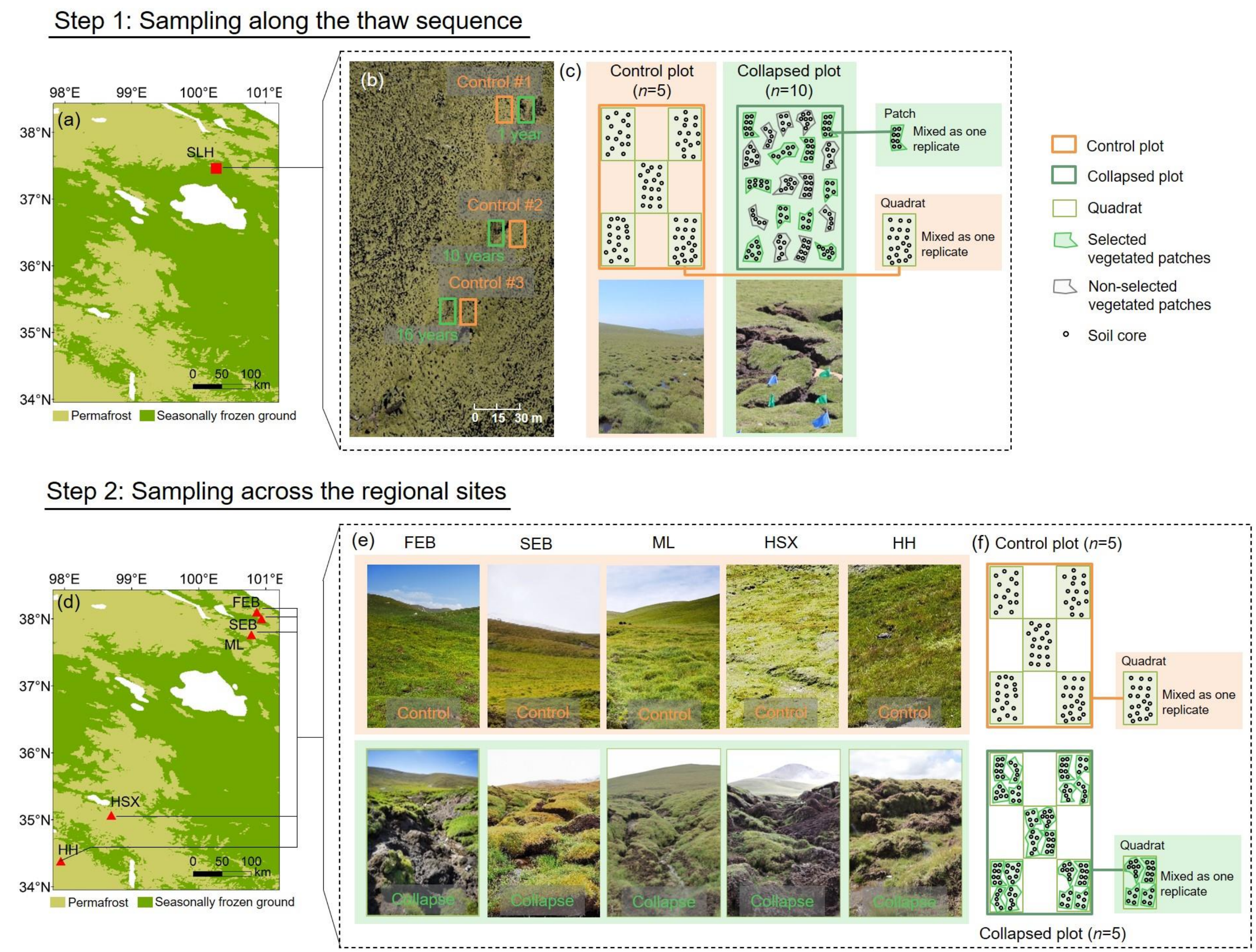
Step 1: Sampling along the thaw sequence
(a)
98°E
99°E
100°E
101°E
38°N
37°N
36°N
35°N
34°N
SLH
0 50 100
km
Permafrost
Seasonally frozen ground
(b)
Control #1
1 year
Control #2
10 years
Control #3
16 years
0 15 30 m
(c)
Control plot
(n=5)
Collapsed plot
(n=10)
Patch
Mixed as one replicate
Quadrat
Mixed as one replicate
Control plot
Collapsed plot
Quadrat
Selected vegetated patches
Non-selected vegetated patches
Soil core
Step 2: Sampling across the regional sites
(d)
98°E
99°E
100°E
101°E
38°N
37°N
36°N
35°N
34°N
FEB
SEB
ML
HSX
HH
0 50 100
km
Permafrost
Seasonally frozen ground
(e)
FEB
SEB
ML
HSX
HH
Control
Control
Control
Control
Control
Collapse
Collapse
Collapse
Collapse
Collapse
(f) Control plot (n=5)
Quadrat
Mixed as one replicate
Quadrat
Mixed as one replicate
Collapsed plot (n=5)

Supplementary Fig. 5. Schematic diagram of soil sampling along the thaw sequence (SLH) and across the regional sites (marked as FEB, SEB, ML, HSX and HH) on the Tibetan Plateau. a, Location of the permafrost thaw sequence based on the map of permafrost distribution on the Tibetan Plateau[3]. b, Image of the thermo-erosion gully captured from coloured LiDAR point cloud data (VZ-400, Riegl, Horn, Austria) with the specific plot distribution along the thaw sequence (photo credit: F.T. Liu). Control #1, Control #2 and Control #3 represent three non-collapsed plots which were paired to collapsed plots occurring for 1 year, 10 years and 16 years, respectively. c, Sampling schematic diagram for the thaw sequence (photo credit: F.T. Liu). d, Distribution of five thermokarst-impacted sites at the regional scale. e, Landscapes of the regional sites with non-collapsed control and collapsed plots (photo credit: Z.L. Li). f, Sampling schematic diagram for the regional sites (drawn by F.T. Liu). SLH, site at Shaliuhe; FEB, the first site at Ebo; SEB, the second site at Ebo; ML, site at Mole; HSX, site at Huashixia; HH, site at Huanghe. Notably, along the thaw sequence, the distance between the collapsed plots occurring for 1 year and those for 10 and 16 years is 80 m and 130 m respectively, while the distance between the collapsed and non-collapsed control plots is less than 1 m. Across the five sites over the regional scale, we set up one paired collapsed and control plots (15 × 10 m) at the end of a gully and in adjacent non-collapsed areas at each site. These five sites were distributed across a 550-km permafrost transect on the Tibetan Plateau. Under our sampling design, there were five replicates in the control plot and ten replicates in the collapsed plots along the thaw sequence. Across the five thermokarst-impact sites over the regional scale, there were five

replicates in both control and collapsed plots. The map images (a and d) were created by authors using ArcMap 10.2 (Environmental Systems Research Institute, Inc., Redlands, CA, USA) / Zou, D., Zhao, L., Sheng, Y., Chen, J., Hu, G., Wu, T., Wu, J., Xie, C., Wu, X., Pang, Q., Wang, W., Du, E., Li, W., Liu, G., Li, J., Qin, Y., Qiao, Y., Wang, Z., Shi, J., and Cheng, G.: A new map of permafrost distribution on the Tibetan Plateau, The Cryosphere, 11, 2527–2542, https://doi.org/10.5194/tc-11-2527-2017, 2017 (https://tc.copernicus.org/articles/11/2527/2017/) / CC BY (https://creativecommons.org/licenses/by/3.0/).

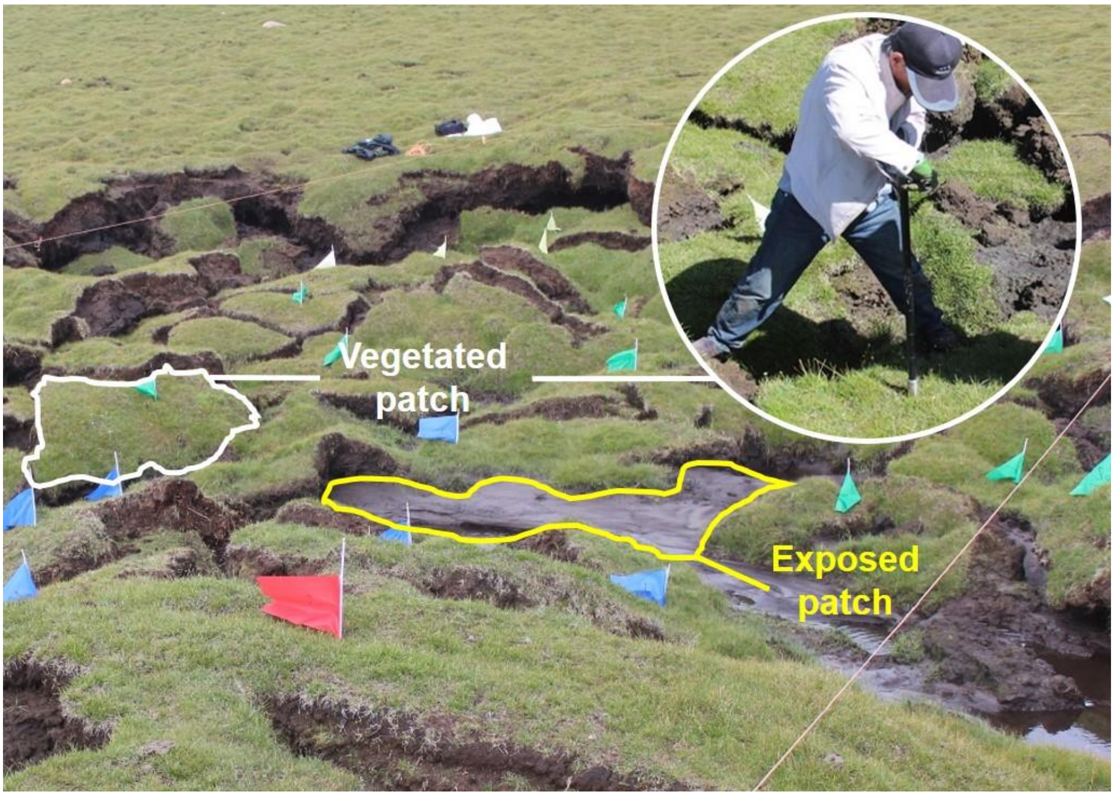

Supplementary Fig. 6. Picture showing vegetated patches (marked with white border) and exposed patches (marked with yellow border) within the collapsed plot across the Tibetan thermokarst-impacted sites (photo credit: F.T. Liu). The inset shows topsoil sampling in vegetated patches (photo credit: F.T. Liu). Notably, to avoid the interference of soil layer mixture, we collected topsoil (0-15 cm) within the vegetated patches (40-60 cm thickness) rather than within the exposed patches, in which soil cores were at least 10 cm away from the edge of vegetated patch.

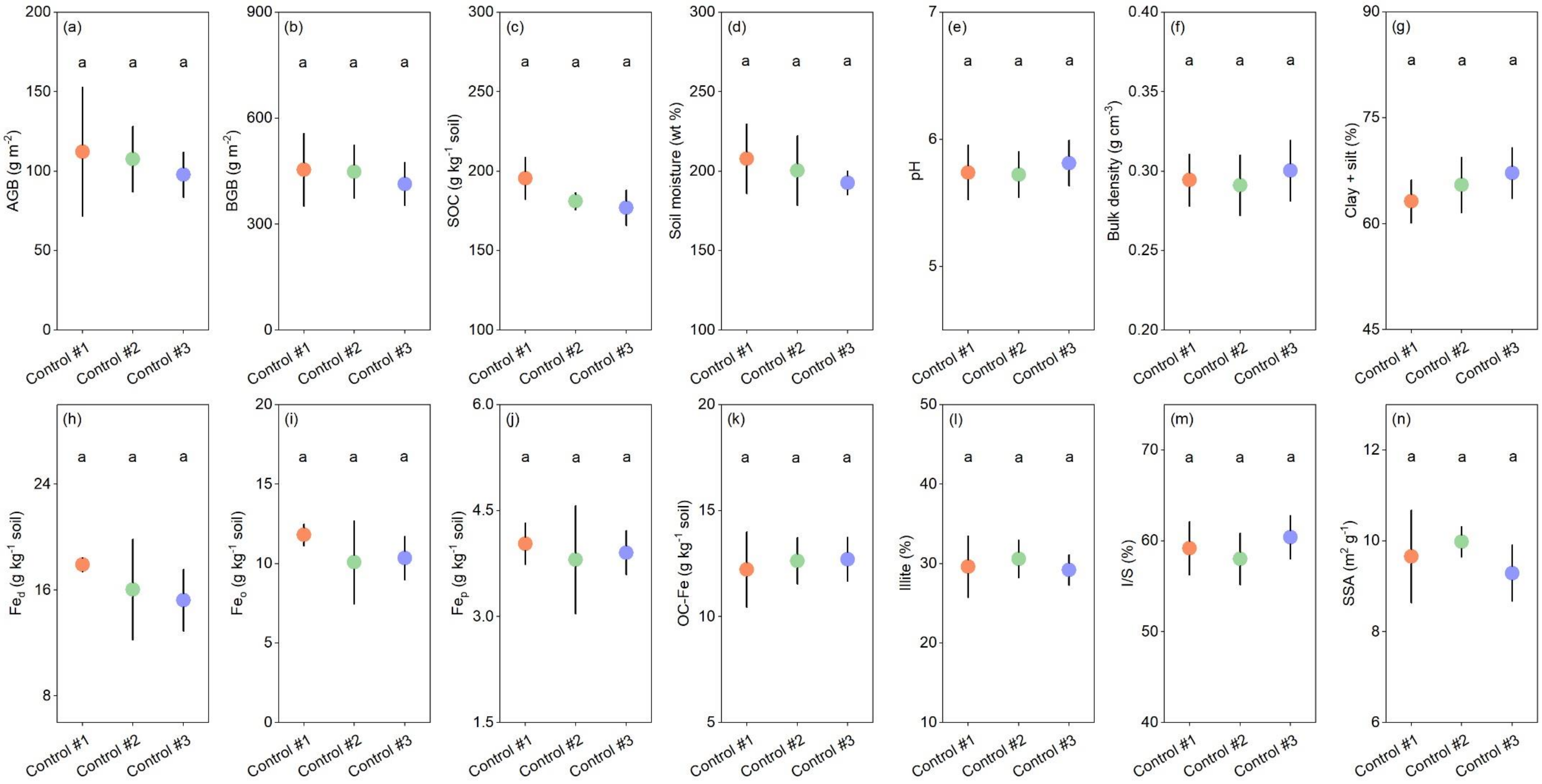

Supplementary Fig. 7. Comparisons of biotic and abiotic properties among three control plots which were adjacent to the corresponding collapsed plots (collapsed for 1 year, 10 years and 16 years) within the thermo-erosion gully. a-n, Comparisons of AGB (a), BGB (b), SOC

(c), soil moisture (d), pH (e), bulk density (f), soil texture (g), Fe_d (h), Fe_o (i), Fe_p (j), OC-Fe (k), illite (l), I/S (m) and SSA (n) between three control plots. Control #1, Control #2 and Control #3 represent three non-collapsed plots which were paired to collapsed plots occurring for 1 year, 10 years and 16 years, respectively (for detailed plot distribution see Supplementary Fig. 5). AGB, aboveground biomass; BGB, belowground biomass; SOC, soil organic carbon; Clay + silt, the percentage of clay and silt; Fe_d, pedogenic Fe oxides; Fe_o, poorly crystalline Fe oxides; Fe_p, organically complexed Fe oxides; OC-Fe, iron-bound organic carbon; I/S, mixed-layer illite/smectite minerals; SSA, specific surface area in mineral-associated organic matter. Error bars represent 95% confidence intervals. Same letters represent no significant differences among the different plots (LSD test, $P > 0.05$). Notably, aboveground and belowground biomass, SOC, soil moisture, pH, bulk density and soil texture were reanalyzed from published data in refs.[1,2], while other parameters were measured in this study.

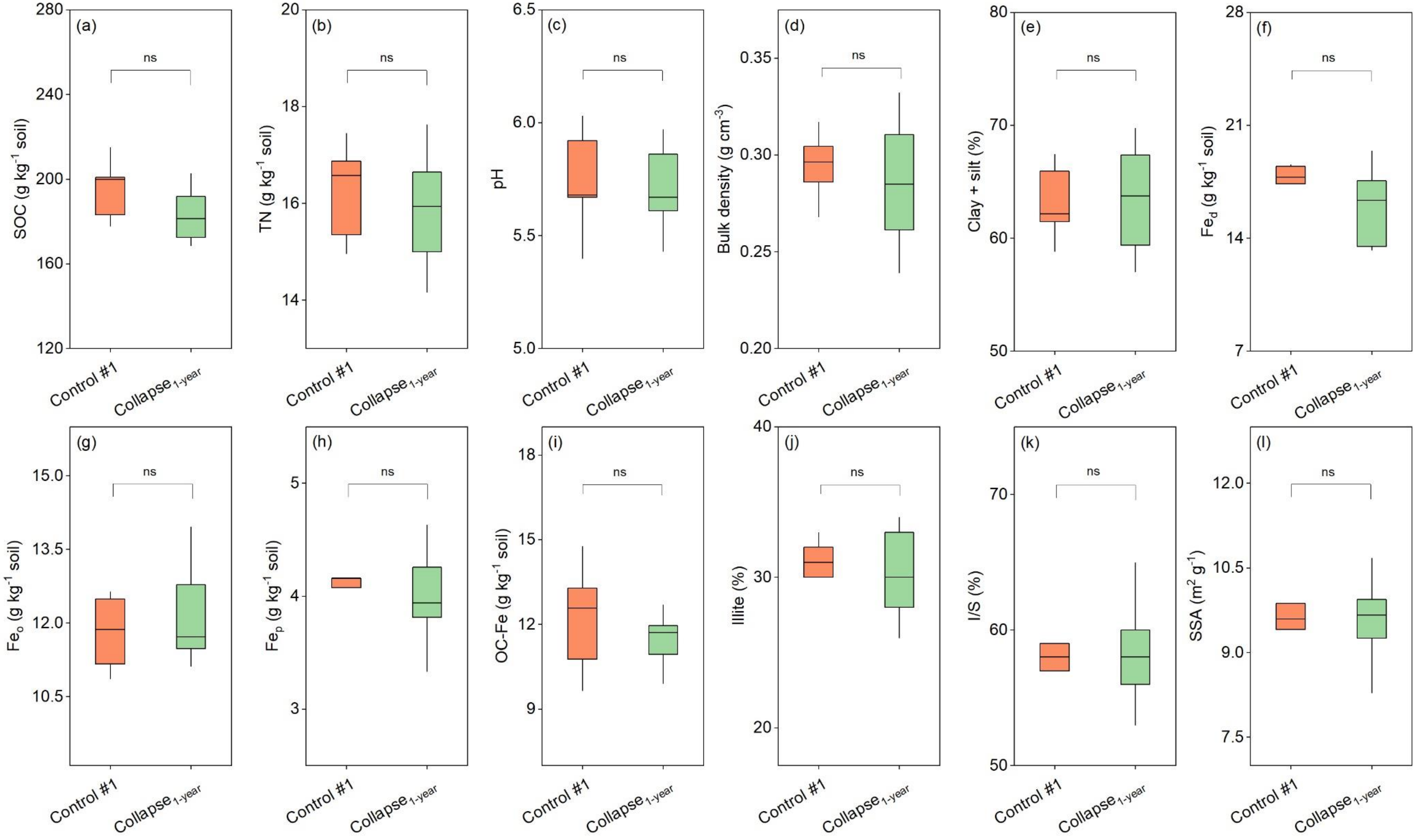
(a)
(b)
(c)
(d)
(e)
(f)
(g)
(h)
(i)
(j)
(k)
(l)
ns
SOC (g kg^{-1} soil)
TN (g kg^{-1} soil)
pH
Bulk density (g cm^{-3})
Clay + silt (%)
Fe_d (g kg^{-1} soil)
Fe_o (g kg^{-1} soil)
Fe_p (g kg^{-1} soil)
OC-Fe (g kg^{-1} soil)
Illite (%)
I/S (%)
SSA (m^2 g^{-1})
Control #1
$Collapse_{1\text{-}year}$

Supplementary Fig. 8. Comparisons of biotic and abiotic properties between non-collapse (control) and collapsed plots (collapsed for 1 year, one stage along the permafrost thaw sequence). a-l, Comparisons of SOC (a), TN (b), pH (c), bulk density (d), soil texture (e), Fe_d (f), Fe_o (g), Fe_p (h), OC-Fe (i), illite (j), I/S (k) and SSA (l) between control and collapsed plots occurring for 1 year. Control #1 and $Collapse_{1\text{-year}}$ denote the paired non-collapsed control and 1-year collapsed plots, respectively (for detailed plot distribution see Supplementary Fig. 5). SOC, soil organic carbon; TN, total nitrogen; Clay + silt, the percentage of clay and silt; Fe_d, pedogenic Fe oxides; Fe_o, poorly crystalline Fe oxides; Fe_p, organically complexed Fe oxides; OC-Fe, iron-bound organic carbon. I/S, mixed-layer illite/smectite minerals; SSA, specific surface area in mineral-associated organic matter. The whiskers denote the 5th and 95th percentiles, and the ends of the boxes represent the 25th and 75th quartiles. ns, insignificant difference (LSD test, $P > 0.05$). Notably, SOC, pH, bulk density and soil texture were reanalyzed from published data in refs.[1,2], while other parameters were measured in this study.

Supplementary Tables

Supplementary Table 1. Mass distribution, carbon concentration and C/N ratios in particulate organic matter (POM), heavy particulate organic matter (HPOM) and mineral-associated organic matter (MAOM) across the thermokarst-impacted sites on the Tibetan Plateau.

Site	Plot	Weight of POM (%)	POM carbon concentration (g kg^{-1} POM)	POM C/N ratio	Weight of HPOM (%)	HPOM carbon concentration (g kg^{-1} HPOM)	HPOM C/N ratio	Weight of MAOM (%)	MAOM carbon concentration (g kg^{-1} MAOM)	MAOM C/N ratio
SLH	Control	3.4 ± 0.4a	346.3 ± 13.6a	19.8 ± 1.7a	34.6 ± 0.8a	236.2 ± 2.5a	12.5 ± 0.1a	51.8 ± 1.6c	148.6 ± 3.9a	10.8 ± 0.2b
	1 year	3.5 ± 0.2a	381.4 ± 9.2a	20.0 ± 0.8a	31.2 ± 1.3b	246.9 ± 4.5a	12.5 ± 0.1a	59.8 ± 1.2b	137.1 ± 4.3b	10.8 ± 0.2b
	10 years	2.8 ± 0.2ab	263.2 ± 17.0b	18.8 ± 1.4ab	30.1 ± 1.4b	233.3 ± 2.8a	12.4 ± 0.1a	61.6 ± 1.6b	129.8 ± 2.9b	10.9 ± 0.1b
	16 years	2.2 ± 0.3b	255.1 ± 12.5b	18.4 ± 0.9b	25.3 ± 1.4c	207.2 ± 15.0b	12.1 ± 0.2a	69.2 ± 1.5a	104.8 ± 4.7c	11.2 ± 0.2a
FEB	Control	1.1 ± 0.1a	201.1 ± 10.9a	22.3 ± 1.1a	20.5 ± 3.2a	171.9 ± 16.1a	13.8 ± 0.2a	67.3 ± 3.8a	89.4 ± 6.1a	11.2 ± 0.1b
	Collapse	0.8 ± 0.1b	170.1 ± 5.8b	21.0 ± 0.5b	14.9 ± 1.1a	118.5 ± 6.6b	13.2 ± 0.3a	76.6 ± 2.1a	86.3 ± 6.4a	11.6 ± 0.2a
SEB	Control	3.5 ± 0.5a	270.8 ± 12.0a	21.8 ± 1.8a	34.8 ± 1.2a	168.3 ± 7.5a	13.3 ± 0.1a	54.7 ± 2.4a	103.1 ± 12.2a	10.9 ± 0.2b
	Collapse	2.1 ± 0.3b	191.5 ± 10.1b	19.6 ± 1.0b	31.3 ± 1.5a	108.0 ±2.3b	12.8 ± 0.2a	56.4 ± 2.0a	95.0 ± 5.5a	11.3 ± 0.1a
ML	Control	3.8 ± 0.4a	289.3 ± 11.0a	24.3 ± 0.6a	28.2 ± 2.6a	225.5 ± 11.2a	13.8 ± 0.3a	61.1 ± 2.8b	126.7 ± 5.0a	11.3 ± 0.3a
	Collapse	1.8 ± 0.4b	236.1 ± 18.1b	22.6 ± 1.4b	23.5 ± 1.6a	167.6 ± 5.9b	13.2 ± 0.2a	70.5 ± 1.2a	95.1 ± 1.0b	11.5 ± 0.3a
HSX	Control	1.2 ± 0.2a	259.4 ± 15.1a	20.0 ± 1.5a	39.2 ± 1.1a	156.6 ± 5.4a	12.9 ± 0.1a	46.8 ± 1.3b	84.2 ± 3.5a	10.3 ± 0.2a
	Collapse	0.8 ± 0.1b	165.2 ± 11.9b	19.1 ± 1.9a	28.9 ± 2.0b	104.7 ± 4.9b	12.3 ± 0.4a	60.5 ± 2.2a	64.7 ± 3.2b	10.1 ± 0.3a
HH	Control	3.1 ± 0.7a	198.3 ± 17.7a	21.1 ± 1.6a	35.7 ± 0.9a	102.6 ± 2.2a	13.4 ± 0.2a	49.1 ± 1.3a	97.8 ± 4.7a	10.9 ± 0.3a
	Collapse	0.9 ± 0.3b	182.3 ± 5.2a	19.2 ± 0.4b	38.7 ± 2.0a	92.1 ± 11.0a	13.4 ± 0.2a	48.1 ± 1.6a	85.5 ± 8.0a	10.6 ± 0.2a

Data are means ± standard errors. SLH, site at Shaliuhe; FEB, the first site at Ebo; SEB, the second site at Ebo; ML, site at Mole; HSX, site at

Huashixia; HH, site at Huanghe. The carbon concentration of POM, HPOM and MAOM means the quantities of carbon normalized to unit POM, HPOM, and MAOM. C/N ratio represent the ratio of organic carbon to total nitrogen. Different letters indicate significant differences between collapse and control plots (LSD test, $P < 0.05$).

Supplementary Table 2. Characteristics of the sampling sites across the Tibetan Plateau.

Site	Coordinates	Elevation (m)	MAT (°C)	MAP (mm)	ALT (m)	Vegetation type	Soil type	Parent material	Mineralogy of clay (< 2 μm)	Thermokarst type
FEB	38.03°N, 100.89°E	3515	2.6	367	0.83	Swamp meadow	Cryosols	SS	Ilt, I/S, Chl, Kao	Thermo-erosion gully
SEB	38.00°N, 100.91°E	3650	2.6	367	0.71	Swamp meadow	Cryosols	SS	Ilt, I/S, Chl, Kao	Thermo-erosion gully
ML	37.76°N,100.80°E	3760	1.4	411	1.10	Swamp meadow	Cryosols	SS	Ilt, I/S, Chl, Kao	Thermo-erosion gully
SLH	37.46°N, 100.28°E	3847	0.1	402	0.86	Swamp meadow	Cryosols	US	Ilt, I/S, Chl, Kao	Thermo-erosion gully
HSX	35.06°N, 98.71°E	4460	-1.0	353	0.82	Swamp meadow	Cryosols	US	Ilt, I/S, Chl, Kao	Thermo-erosion gully
HH	34.38°N, 97.95°E	4707	-3.1	436	0.78	Swamp meadow	Cryosols	SS	Ilt, I/S, Chl, Kao	Thermo-erosion gully

FEB, the first site at Ebo; SEB, the second site at Ebo; ML, site at Mole; SLH, site at Shaliuhe; HSX, site at Huashixia; HH, site at Huanghe; MAT, mean annual temperature; MAP, mean annual precipitation; ALT, active layer thickness; SS, siliciclastic sedimentary; US, unconsolidated sediments; Ilt, illite; I/S, mixed-layer illite/smectite; Chl, chlorite; Kao, kaolinite. The MAT and MAP data were derived from the National Meteorological Information Center (http://data.cma.cn). Soil type was classified based on the World Reference Base for Soil Resources[4]. Parent material was identified according to the new global lithological map database GLiM[5].

Supplementary Table 3. Mean value and coefficient of variation of parameters from control plots (non-collapsed areas; for detailed plot information see Supplementary Fig. 5) across the five thermokarst-impacted sites over the regional scale.

Site	SOC ($g\ kg^{-1}$ soil)		Soil moisture (wt %)		pH		Clay+Silt (%)		Fe_d ($g\ kg^{-1}$ soil)		Fe_o ($g\ kg^{-1}$ soil)		Fe_p ($g\ kg^{-1}$ soil)		Illite (%)		I/S (%)	
	Mean	CV(%)	Mean	CV(%)	Mean	CV(%)	Mean	CV(%)	Mean	CV(%)	Mean	CV(%)	Mean	CV(%)	Mean	CV(%)	Mean	CV(%)
FEB	98.3	6.1	102.3	11.1	6.6	1.7	62.4	2.7	15.3	8.0	9.6	6.8	2.9	13.1	28.2	11.0	64.2	5.6
SEB	129.5	11.2	153.0	14.4	6.6	4.6	68.3	4.1	13.2	10.6	8.5	13.2	1.9	11.6	41.8	8.5	47.8	6.8
ML	153.0	12.3	131.3	11.3	5.7	3.7	72.6	3.0	19.1	3.7	11.0	5.7	3.1	3.3	28.0	13.6	62.4	8.3
HSX	107.3	4.8	134.7	14.1	5.8	3.6	45.2	2.5	10.3	6.8	5.3	9.1	1.7	13.2	41.4	7.4	43.2	8.7
HH	90.6	7.6	139.6	7.3	6.4	5.4	42.3	7.9	12.3	14.2	6.2	13.1	2.2	11.2	34.0	5.5	54.2	5.1

FEB, the first site at Ebo; SEB, the second site at Ebo; ML, site at Mole; HSX, site at Huashixia; HH, site at Huanghe; CV, coefficient of variation (%); SOC, soil organic carbon; Clay+silt, the percentage of clay and silt; Fe_d, pedogenic Fe oxides; Fe_o, poorly crystalline Fe oxides; Fe_p, organically complexed Fe oxides; I/S, mixed-layer illite/smectite.

Supplementary References

1. Chen, L. et al. Nitrogen availability regulates topsoil carbon dynamics after permafrost thaw by altering microbial metabolic efficiency. *Nat. Commun.* **9**, 3951 (2018).
2. Liu, F. et al. Reduced quantity and quality of SOM along a thaw sequence on the Tibetan Plateau. *Environ. Res. Lett.* **13**, 104017 (2018).
3. Zou, D. et al. A new map of permafrost distribution on the Tibetan Plateau. *Cryosphere* **11**, 2527-2542 (2017).
4. IUSS Working Group WRB: World reference base for soil resources 2014. International soil classification system for naming soils and creating legends for soil maps (FAO, Rome, 2014).
5. Hartmann, J. & Moosdorf, N. The new global lithological map database GLiM: A representation of rock properties at the Earth surface. *Geochem. Geophys. Geosyst.* **13**, Q12004 (2012).
