# Supplementary material for: Divergent changes in particulate and mineral-associated organic carbon upon permafrost thaw
Source: Nat Commun. 2022 Aug 29;13:5073. doi: 10.1038/s41467-022-32681-7 (PMC9424277; doi:10.1038/s41467-022-32681-7)
Supplement: Supplementary file 3 — Description of Additional Supplementary Files [file 41467_2022_32681_MOESM3_ESM.pdf]

File name: Supplementary Data 1

Description: Data of soil carbon fractions, vegetation biomass, edaphic variables and soil minerals used in this study.
